# Effective and cheap removal of leukocytes and platelets from *Plasmodium vivax *infected blood

**DOI:** 10.1186/1475-2875-8-115

**Published:** 2009-06-02

**Authors:** Kanlaya Sriprawat, Supaporn Kaewpongsri, Rossarin Suwanarusk, Mara L Leimanis, Usa Lek-Uthai, Aung Pyae Phyo, Georges Snounou, Bruce Russell, Laurent Renia, François Nosten

**Affiliations:** 1 Shoklo Malaria Research Unit, Mae Sod, PO Box 46, Tak 63110, Thailand; 2 Singapore Immunology Network (SIgN), IMMUNOS Building 3-4, BIOPOLIS, 8A Biomedical Grove, 138648, Singapore; 3 Department of Parasitology and Entomology, Faculty of Public Health, Mahidol University, 420/1 Rajvithi Road, Rajthewee, Bangkok 10400, Thailand; 4 Département de Parasitologie, INSERM UMR S 945, Hôpital Pitié-Salpêtrière, Paris, France; 5 Faculté de Médecine Pitié-Salpêtrière, Université Pierre et Marie Curie, 4 place jussieu, 75005, Paris, France; 6 Department of Microbiology, Laboratory of Molecular and Cellular Parasitology, Faculty of Medicine, National University of Singapore, 5 Science Drive 2, 117597, Singapore; 7 Nano Biomechanics Laboratory, Division of Bioengineering and Department of Mechanical Engineering, National University of Singapore, 10 Kent Ridge Crescent, 119260, Singapore; 8 Medicine, Nuffield Department of Clinical Medicine, University of Oxford, CCVTM, Oxford, OX3 7LJ, UK; 9 Faculty of Tropical Medicine, Mahidol University, 420/6 Rajvithi Road, Bangkok, 10400, Thailand

## Abstract

**Background:**

Investigations of *Plasmodium vivax *are restricted to samples collected from infected persons or primates, because this parasite cannot be maintained in *in vitro *cultures. Contamination of *P. vivax *isolates with host leukocytes and platelets is detrimental to a range of *ex vivo *and molecular investigations. Easy-to-produce CF11 cellulose filters have recently provided us with an inexpensive method for the removal of leukocytes and platelets. This contrasted with previous reports of unacceptably high levels of infected red blood cell (IRBC) retention by CF11. The aims of this study were to compare the ability of CF11 cellulose filters and the commercial filter Plasmodipur at removing leukocyte and platelet, and to investigate the retention of *P. vivax *IRBCs by CF11 cellulose filtration.

**Methods and Results:**

Side-by-side comparison of six leukocyte removal methods using blood samples from five healthy donor showed that CF11 filtration reduced the mean initial leukocyte counts from 9.4 × 10^3 ^per μl [95%CI 5.2–13.5] to 0.01 × 10^3 ^[95%CI 0.01–0.03]. The CF11 was particularly effective at removing neutrophils. CF11 treatment also reduced initial platelet counts from 211.6 × 10^3 ^per μl [95%CI 107.5–315.7] to 0.8 × 10^3 ^per μl [95%CI -0.7–2.2]. Analysis of 30 *P. vivax *blood samples before and after CF11 filtration showed only a minor loss in parasitaemia (≤ 7.1% of initial counts). Stage specific retention of *P. vivax *IRBCs was not observed.

**Conclusion:**

CF11 filtration is the most cost and time efficient method for the production of leukocyte- and platelet-free *P. vivax*-infected erythrocytes from field isolates.

## Background

The emergence of drug resistance[1] and the renewed awareness of severity in vivax malaria[2] is spurring efforts to better understand this important pathogen. However, exploiting the recently published genome[3] and transcriptome[4] of *Plasmodium vivax *still relies on the use of infected blood samples collected from patients or experimentally infected simians, because it is not yet possible to continuously culture this parasite.

Removal of leukocytes and other components from infected blood samples is an important prerequisite for a number of investigations. Sequencing the parasite's genome can be significantly hampered by the relatively large quantities of host DNA present in white blood cells. Furthermore, it has been shown that the leukocytes present in samples can phagocytise, damage and potentially destroy malaria parasites under *ex vivo *investigations[5,6]. Antimicrobial and biochemical studies of infectious diseases may also be confounded by the significant metabolic activity of leukocytes in the sample of interest. Recently, the bioethical regulations in many countries mandate the removal of human leukocytes from infected blood samples, before transfer to other countries, in order to curb the possibility of unauthorized human genomic research. Other blood components are best removed from the collected blood before *in vitro *testing. Platelets often bind to and degranulate on contact with infected red blood cells (IRBCs) adversely affecting the parasite's *ex vivo *development[7].

The wide range of leukocyte removal techniques that have been developed since the 1950s, are mostly based on differential centrifugation or on column filtration. Differential density centrifugation using sucrose solutions, Percoll™, Nycodenz™, Ficoll™ and Lymphoprep™ (Greiner Bio-One^®^) are particularly useful when a viable leukocyte fraction is needed for subsequent immunological investigations. However, these methods are particularly time consuming. It is generally agreed that column filtration methods are a more practical, rapid and effective method for the removal of leukocytes [8-10]. Custom-made CF11 cellulose powder columns are significantly less expensive than commercial filters such as Plasmodipur, however, it has been reported that CF11 retains some IRBCs and in particular mature asexual stages (trophozoite and schizont) of *P. vivax*[11]. This contrasts with observations made in recent studies where the use of CF11 cellulose filters with *P. vivax *samples did not result in a high loss of mature stages after filtration [12-14].

The two aims of this study were to compare the efficacy of leukocyte and platelet removal efficacy of CF11-based filters and of Plasmodipur, and to investigate whether stage specific retention of *P. vivax *IRBCs occurs through CF11 cellulose filtration.

## Methods

### CF11 column construction

A 10 ml syringe (with the plunger removed) was tipped with two 1 cm^2 ^pieces of Grade 105 lens cleaning tissue (Whatman^®^). The tissue was placed in the syringe so as to cover the outlet lumen. Only syringes with a centred rather than an offset outlet should be used. Ten ml of loosely packed of CF11 cellulose powder (Whatman^®) ^were added to the syringe and then packed down to ~5.5 ml of packed cellulose. The tip and bottom of the syringe were covered with aluminium foil, and then autoclaved. When ready to use, the CF11 column was wetted with ~5 ml of isotonic phosphate buffered saline (PBS) solution (without Ca^2+ ^and Mg^2+^, pH 7.3).

### Sample collection and processing

Due to the limited quantity of *P. vivax *IRBC healthy donor blood was used for the initial side-by-side comparisons of leukocytes and platelet removal methods. Ten ml of whole blood were collected onto Lithium heparin from five healthy donors by venepuncture. The whole blood was centrifuged at 500 g for 5 min at room temperature. The plasma supernatant was removed, but the buffy coat fraction containing white blood cells (WBC) was added back to the red blood cell (RBC) pellet. The RBC+WBC mix from each donor was divided into five two ml portions to which an equal volume of RPMI was added with gentle mixing. The first tube was used as the control for this study. The 2^nd ^tube was centrifuged again as above and the PBS supernatant and buffy coat carefully removed from the packed RBC, this tube was the 'Buffy Coat Removal' treatment. The 3^rd ^tube was loaded into a five ml syringe (the plunger removed) and mounted onto a Plasmodipur™ filter (Euro-Diagnostica^®^) that was pre-rinsed with a sterile PBS solution. Then gentle pressure was applied to the syringe attached to the Plamodipur unit and the filtered RBC/PBS (50% haematocrit) mix was collected as the 'Plasmodipur' treatment. The 4^th ^and 5^th ^tubes were added to PBS-wetted CF11 columns. The filtered 4^th ^sample was then collected as the 'CF11' treatment. The filtered 5^th ^sample was then added to another unused CF11 column (pre-wetted with 5 ml of PBS). The double filtered 5^th ^sample was the 'CF11x2' treatment. In addition to the above samples, three more healthy volunteers were recruited, and 8 ml of whole blood were collected onto Lithium heparin. This blood was processed as above and divided into four 2 ml samples. The first and 2^nd ^tubes were processed as described above for the control and the 'CF11' treatments, respectively. The contents of the 3^rd ^tube were layered over 2 ml of Lymphoprep™ (Greiner Bio-One^®^) and centrifuged at 600 g for 20 min at 20°C, the leukocytes that banded at the interface were removed using a pipette, and the supernatant RBC fraction was the 'Lymphoprep' treatment. The 4^th ^tube was processed as above for the 'Lymphoprep' treatment, but then subsequently processed using the 'Plasmodipur' treatment protocol described above. This final sample was called the 'Lymphoprep and Plasmodipur' treatment.

Please note; unless specifically stated, the term 'CF11 filtration' involved only a single CF11 filter, as opposed to 'CF11x2' which involved processing the sample using two CF11 columns.

Complete blood counts of the control samples and the suspensions obtained from the six treatments were conducted using an Automated Hematology Analyzer (Model pocH-100i, Sysmex Company) and by microscopic examination (x100 oil immersion) of Giemsa-stained thick (250 fields) and thin smears (450 fields). The data from the thin smears were used for differential lymphocyte counts.

### Effect of CF11 filtration on *P. vivax *isolates

Thick and thin smears were routinely made for the pre- and post-CF11 filtration from each of the *P. vivax *isolates collected, prior to *ex vivo *drug sensitivity testing at the Shoklo Malaria Research Unit (SMRU), Mae Sod, Thailand. The pre- and post-CF11 filtration, thick and thin smears from 30 randomly selected isolates collected during 2008 were examined as follows. Parasitaemias were determined from the number of IRBC per ten 100× oil immersion fields (200 RBC per field) on the thin film. The percentage of early (ring-like parasites with a single chromatin dot) and mature (amoeboid-like cytoplasm or presence of haemozoin) asexual stages was determined from examining 200 parasites in the thick smears under 100× oil immersion. Due to their very low numbers in the pre- and post-filtration smears schizonts (parasites with 3 or more chromatin dots and haemozoin) were combined with the mature stage count. Gametocyte counts were too low for statistical comparison.

Parametric analysis of non-paired data was calculated using one-way analysis of variance. Non-parametric analysis of the paired data was performed using Wilcoxon or Friedman's tests and post-hoc analysis using Dunn's test (GraphPad Prism 5.01).

The clinical IRBC samples examined in this study were collected under the following ethical guidelines in the approved protocol OXTREC 027-05 (University of Oxford, Centre for Clinical Vaccinology and Tropical Medicine, UK)

## Results and discussion

### Cost- and time-effective removal of leukocytes

**Figure 1:**
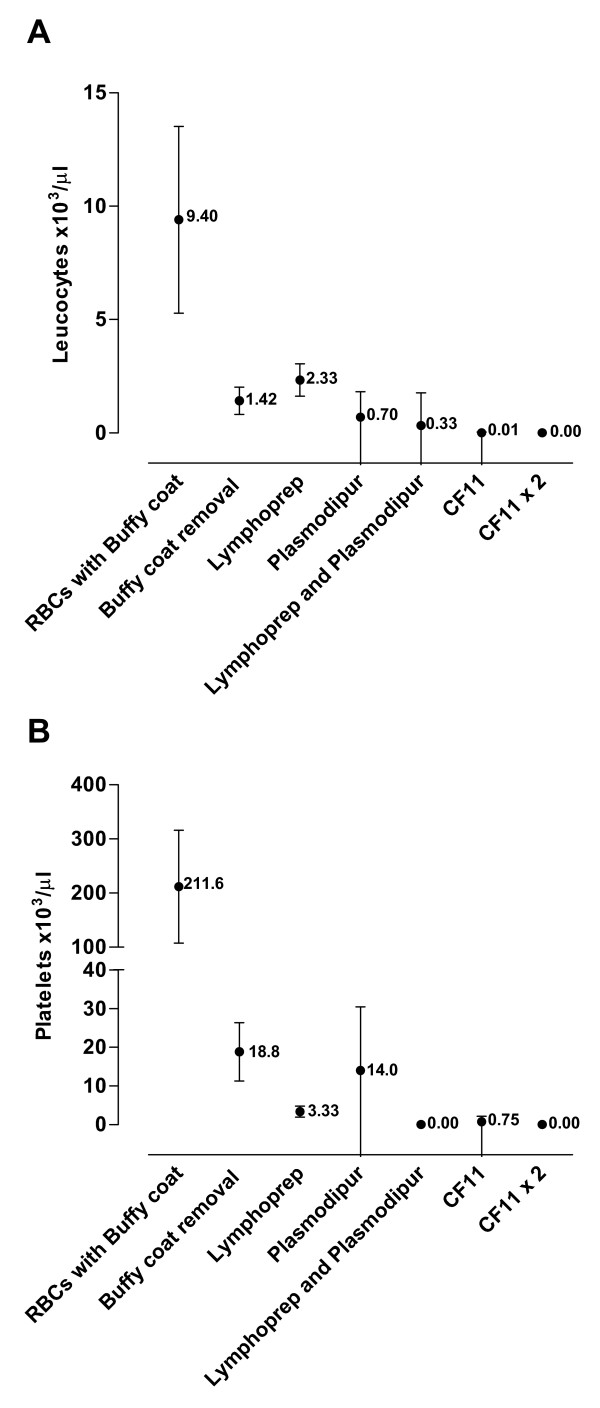
**The comparative efficacy of six methods for removing leukocytes (A) and platelets (B) from five healthy donor blood samples (repeated measures) prior to buffy coat removal**. Leukocyte counts and platelet mean counts × 10^3^μl of whole blood [+/-95% CI]; analysis by Automated Hematology Analyzer (Model pocH-100i, Sysmex Company).

**Figure 2:**
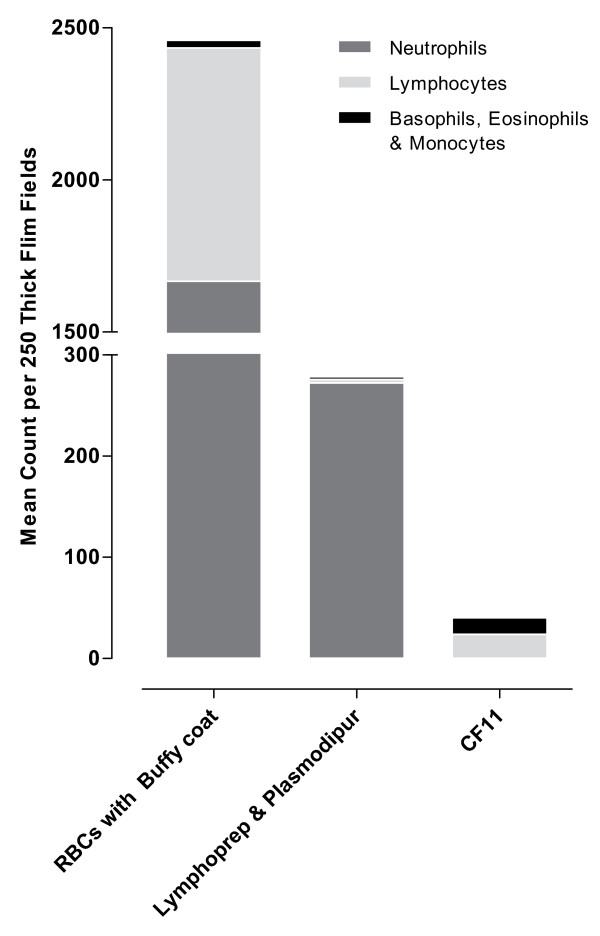
**Comparison of leukocyte composition of five healthy donor blood samples before and after treatment with Lymphoprep and Plasmodipur and CF11 methods**. Leukocyte counts are expressed as Mean Count per 250 Thick Films (100× oil immersion).

Plasmodipur filters were the most rapid method for the filtration of donor blood with ~10 min per isolate as compared to the ~20 min per isolate required for CF11 filtration. A Plasmodipur filtering unit costs ~50–55 USD while a single CF11 column could be made at a total cost of ~1 USD. The effectiveness of the two types of columns to remove leukocytes and platelets from clinical blood samples was then assessed (Figure 1A and 1B). Filtration on CF11 and Plasmodipur reduced the mean initial leukocyte counts from 9.4 × 10^3 ^per μl [95%CI 5.2–13.5] to 0.01 × 10^3 ^[95%CI 0.01–0.03] and 0.7 × 10^3 ^per μl [95%CI 0.4–1.8] respectively (Figure 1A). Filtration on CF11 was particularly effective at removing platelets, reducing the platelet count from 211.6 × 10^3 ^per μl [95%CI 107.5–315.7] to 0.8 × 10^3 ^per μl [95%CI -0.7–2.2], a significantly greater platelet reduction when compared to the mean platelet count of the Plasmodipur filtrate which was 14.0 × 10^3 ^per μl [95%CI -2.3–30.5]) (p = 0.02). Only by pre-processing the blood sample with Lymphoprep prior to Plasmodipur filtration was it possible to reduce the mean leukocyte (0.3 × 10^3 ^per μl [95%CI -1.1–1.8]) and platelet (0.0 × 10^3 ^per μl) contamination to the same levels as that obtained by CF11 filtration. However, pre-treatment with Lymphoprep adds another 50 mins and a further ~8–9 USD to procedure, thus negating the benefits of the relatively speedier Plasmodipur filtration. It was interesting to note that the composition of the contaminating leukocytes in the suspension obtained after Lymphoprep/Plasmodipur or CF11 filtration differed greatly. Leukocyte contamination in the Lymphoprep/Plamodipur filtrate was predominantly neutrophils (98%) with the remainder made up of lymphocytes and basophils/monocytes (Figure 2). By contrast, no neutrophils were found in the CF11 filtrate where the small number of contaminating leukocytes was either lymphocytes (59%) or basophils/eosinophils (41%). The specific removal of neutrophils by CF11 is of particular advantage in *ex vivo *culture, where neutrophils readily phagocytise maturing IRBCs [6].

### The effect of CF11 filtration on *P. vivax *parasite numbers and stages

**Figure 3:**
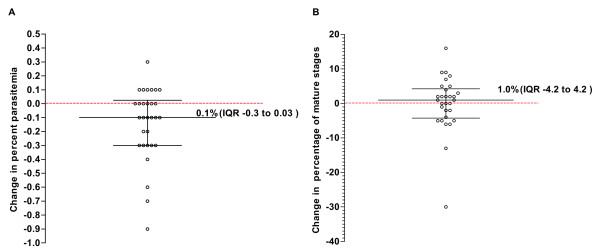
**The effect of CF11 filtration on the parasitaemia (A) and developmental stage composition (B) of 30 Thai *Plasmodium vivax *isolates**. The effect of filtration is expressed as a change in the percentage parasitaemia (IRBC per 10 thin film fields at 100× oil immersion) or percentage mature *P. vivax *erythrocytic stages [(mature stages/(early stages + mature stages)*100)] relative to the blood stages prior to CF11 filtration (no change denoted by the red broken line).

Filtration of *P. vivax *infected isolates resulted in median parasitaemia change of -0.1% [IQR -0.3–0.03] (Figure 3A). This corresponds to a 7.14% reduction in IRBCs from the initial median parasitaemia of 0.7% [IQR 0.38–1.12] to a post filtration median parasitaemia of 0.65% [IQR 0.30–1.0] (*p *= 0.01). The magnitude of IRBC loss was much less than the major losses previously reported for CF11 filtration, namely from a starting parasitaemia of 0.37% to 0.18% in the filtrate for one sample and 0.43% to 0.01% for the other, representing a 52% and 98% reduction in parasitaemia respectively [11]. It was further reported that CF11 selectively retained mature trophozoites and schizonts, with only ring stages passing through the CF11 unimpeded[11]. In this study we failed to observe any selective retention of mature *P. vivax *stages, the median pre- and post-filtration composition of late stages in the *P. vivax *samples was 17% and 19% respectively. The median change in the composition of late stages pre- and post-filtration was an increase of 1.0% [IQR -4.2 to 4.2] (*p *= 0.06) (Figure 3B). This is consistent with our experience with routine use of CF11 filtration of *P. vivax *isolate where the loss of mature forms were not noted[12,13,15], and where CF11 filtration did not effect *P. vivax *maturation of early or late stage trophozoites.

It should be noted that equally satisfactory removal of leukocytes and platelets, with little loss of parasites, were obtained when the PBS solution was used to wash and resuspend the blood and the CF11 columns was replaced by RPMI or McCoy's 5A medium (GIBCO^®^)

## Conclusion

Custom-made CF11 filtration provides a cost effective method for the removal of leukocytes from *P. vivax *clinical isolates. The small, non stage specific, reduction in parasitaemia due to CF11 filtration has done little to hamper its application to a range of recent *P. vivax *studies [12-14,16], notably the recent report on the *P. vivax *transcriptome[4]. Although the report of high parasite losses due to CF11 filtration were only based on two samples [11], the magnitude of the losses was substantial. It is unlikely that variations in the quality of the CF11 powder account for the discrepancy between this observation and ours. The composition of the buffers used to resuspend and wash the blood samples and the column might, on the other hand, substantially modify the binding properties of the CF11 powder to different cell types. This is evident from the first report of *P. vivax *retention by CF11 [17] where the mere addition of 2 mM EDTA improved the recovery of IRBCs. Furthermore, in our experience the quantity of CF11 powder per ml of whole blood, the degree of column compaction, as well as the amount and extent of washing, influence the efficacy of the protocol. If the 'leukocyte free' status of the IRBC preparation is a priority, we recommend the CF11x2 protocol (Figure 1A). Notwithstanding the detailed description we provide for our protocol, we strongly recommend that the procedure be validated on test samples before its routine adoption.

**Figure 4:**
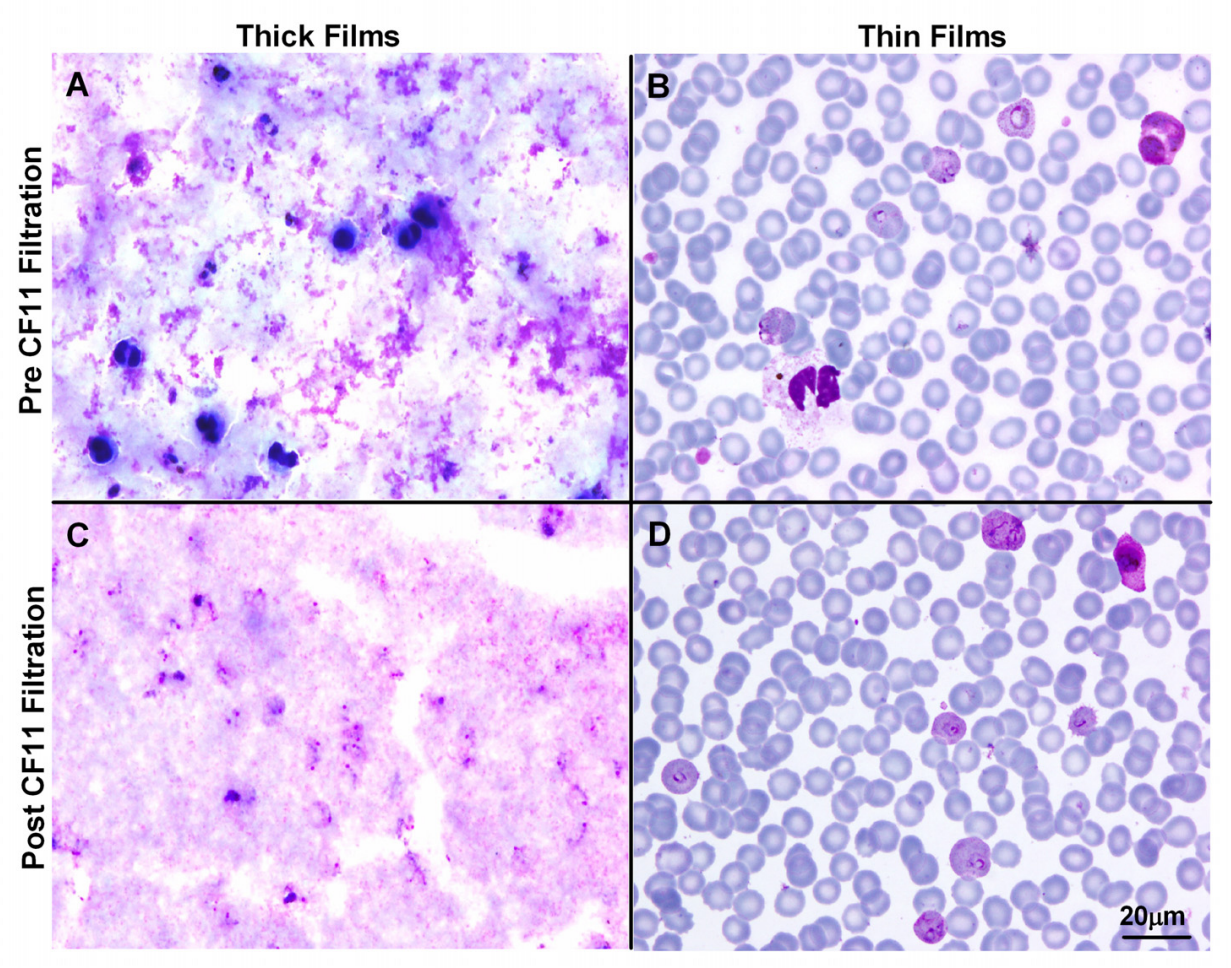
**The effect of CF11 filtration on thick and thin Giemsa stained films**. Photomicrographs of thick (A&C) and thin (B&D) films before and after CF11 filtration (100× oil immersion, Scale bar = 20 μm).

An additional benefit of WBC and platelet filtration, irrespective of the method chosen, is that it aids in the microscopic examination of thick films. Thick films made from filtered isolates produce clear 'noise-free' thick films (Figure 4). This may also be crucial in the successful development of digital recognition software for automating parasite counts [18].

## Competing interests

The authors declare that they have no competing interests.

## Authors' contributions

KS, SK and RS helped design this study, optimized and conducted the experiments, microscopy, and drafted the manuscript. UL and MLL coordinated the field studies, sample collection, and provided intellectual guidance though out this study. BR conceived the study, designed and participated in the experiments, was the cross check microscopist, responsible for photomicrographs and figure production, and initial drafting of the manuscript. GS provided invaluable intellectual input through out the study and significant contributions to the production of the final manuscript. APP and FN coordinated the clinical support and ethical approval. LR and FN jointly directed the study as a whole, providing major intellectual guidance though out this study and during manuscript preparation. All authors read and approved the final manuscript.
